# Correction: Screening potential anti-osteoarthritis compounds using molecular docking based on MAPK and NFκB pathways and validating their anti-osteoarthritis effect

**DOI:** 10.1371/journal.pone.0324192

**Published:** 2025-05-14

**Authors:** Tian-Wang Zhu, Yu Zheng, Rui-Xin Li

The caption for Fig 1 is incorrect. Please see the correct Fig 1 caption here.

**Fig 1.** The roles of the MAPK and NFκB pathways in the onset and progression of osteoarthritis.

The following information should have been the last paragraph of Introduction section: Molecular docking is a powerful computational technique used to simulate the interaction between a compound and a protein to evaluate their binding potential. As a strong competitor to high-throughput omics, this technique has been widely used in drug discovery [10]. However, to our knowledge, no studies focused on screening anti-osteoarthritis compounds using molecular docking. This study aimed to screen potential anti-osteoarthritis compounds by docking with core human proteins in the MAPK and NFκB pathways, analyze their drug-likeness, pharmacokinetics, bioactivity, and toxicity, and test their cytotoxicity and anti-osteoarthritis effect on mouse chondrocytes.

## References

[1] Zhu T-W, Zheng Y, Li R-X. Screening potential anti-osteoarthritis compounds using molecular docking based on MAPK and NFκB pathways and validating their anti-osteoarthritis effect. PLoS One. 2025;20(3): e0319686. doi: 10.1371/journal.pone.0319686 40131994 PMC11936181

