# Supplementary material for: Screening potential anti-osteoarthritis compounds using molecular docking based on MAPK and NFκB pathways and validating their anti-osteoarthritis effect
Source: PLoS One. 2025 Mar 25;20(3):e0319686. doi: 10.1371/journal.pone.0319686 (PMC11936181; doi:10.1371/journal.pone.0319686)
Supplement: S1 Specification — (PDF) [file pone.0319686.s001.pdf]

一氧化氮（NO）含量检测试剂盒说明书（酶法测定总 NO）

可见分光光度法

注意：本产品试剂有所变动，请注意并严格按照该说明书操作。

货号：AC10317

规格：50T/48S

产品组成：使用前请认真核对试剂体积与瓶内体积是否一致，有疑问请及时联系本公司工作人员。

试剂名称	规格	保存条件
提取液	液体 60 mL×1 瓶	2-8℃保存
试剂一	粉剂×1 支	-20℃保存
试剂二	粉剂×1 支	-20℃保存
试剂三	粉剂×2 支	-20℃保存
试剂四	液体 3 mL×1 瓶	2-8℃保存
试剂五	液体 50μL×1 支	2-8℃保存
显色液 A 液	液体 15 mL×1 瓶	2-8℃保存
显色液 B 液	液体 15 mL×1 瓶	2-8℃保存
澄清剂	粉剂×1 瓶	2-8℃保存
标准品	液体 1mL×1 支	2-8℃保存

溶液的配制：

1、 试剂一：临用前加入 1.8 mL 蒸馏水，-20℃分装保存两周，避免反复冻融；
2、 试剂二：临用前加入 1mL 蒸馏水，-20℃分装保存 4 周，避免反复冻融；
3、 试剂二工作液：临用前根据样本量按试剂二：蒸馏水=10μL：590μL（15T）的比例配制，当天用完；
4、 试剂三：临用前取一支加入 550μL 蒸馏水溶解，-20℃分装保存两周，避免反复冻融；
5、 试剂五：临用前根据样本数量按照试剂五（V）：蒸馏水（V）=10μL：450μL（11T）的比例配制试剂五溶液，现用现配；
6、 显色液：临用前根据样本数量按照显色液 A 液：显色液 B 液=1:1 充分混匀，现配现用；
7、 澄清剂：临用前加入 15mL 蒸馏水，可震荡或 50℃加热促进溶解。此溶液为饱和溶液，取上清使用即可，2-8℃可保存 12 周；
8、 标准液：10μmol/mL 亚硝酸钠。临用前取 20μL 10μmol/mL 标准液，加入 780μL 蒸馏水，配制成 0.25μmol/mL 标准液，再取 0.25μmol/mL 标准液 50μL 和蒸馏水 450 μL 混合配制成 0.025μmol/mL 标准溶液。

产品说明：

一氧化氮（Nitric Oxide，NO）是一种极不稳定的生物自由基，分子小，结构简单，常温下为气体，微溶于水，具有脂溶性，可快速透过生物膜扩散，作为一种新型的生物信使分子，在细胞间及细胞内发挥传递信号的作用。其广泛分布于生物体内各组织中，特别是神经组织中。在机体神经、循环、呼吸、消化、泌尿生殖等系统中也起着十分重要的作用。

NO在体内或水溶液中极易氧化生成NO_2^-和NO_3^-，本法利用硝酸还原酶特异性将NO_3^-还原成NO_2^-，在酸性条件下，NO_2^-与重氮盐磺酸胺生成重氮化合物，进一步与萘基乙烯基二胺偶合，产物在550nm处有特征吸收峰，测定其吸光值，可以计算NO含量。

$$NO \longrightarrow NO_3^-$$

$$NO_3^- + NADPH \xrightarrow{\text{Nitrate Reductase}} NO_2^- + NADP^+$$

$$NO_2^- + \text{4-Aminobenzenesulfonamide} \xrightarrow{H^+} \text{Diazonium Salt}$$

$$\text{Diazonium Salt} + \text{N-(1-Naphthyl)-Ethylenediamine} \longrightarrow \text{Red Azo Dyes (550nm)}$$

注意：实验之前建议选择 2-3 个预期差异大的样本做预实验。如果样本吸光值不在测量范围内建议稀释或者增加样本量进行检测。

需自备的仪器和用品：

可见分光光度计、低温离心机、分析天平、水浴锅/恒温培养箱、1mL玻璃比色皿、可调式移液枪、研钵/匀浆器/细胞超声破碎仪、冰和蒸馏水。

操作步骤：

一、样本处理（可适当调整待测样本量，具体比例可以参考文献）

1. 组织样本：按质量（g）：提取液体积（mL）1 : 5~10 比例加入提取液（建议称取 0.2g 样本，加入 1.0mL 提取液），冰浴匀浆后，于 4℃，12000rpm，离心 15min，弃沉淀，取上清液置于冰上待测。
2. 细菌/细胞样本：按细菌/细胞数量（10^4）：提取液体积（mL）500~1000 : 1 的比例加入提取液（建议 1000 万细菌/细胞加入 1.0mL 提取液），冰浴超声破碎细菌/细胞（功率 200w，超声 3s，间隔 7s，总时间 5min），然后于 4℃，12000rpm，离心 15min，弃沉淀，取上清液置于冰上待测。
3. 液体样本：直接测定。若液体有浑浊则离心取上清测定。

二、测定步骤

1．可见分光光度计预热30min以上，调节波长至550nm，蒸馏水调零。

2．操作表：

试剂名称（μL）	测定管	标准管	空白管
样本	240	-	-
0.025μmol/mL标准液	-	240	-
蒸馏水	-	160	400
试剂一	20	-	-
试剂二工作液	40	-	-
试剂三	20	-	-
混匀，37℃反应120min		-	-
试剂四	40	-	-
试剂五	40	-	-
混匀，37℃反应30min		-	-
显色液	400	400	400
混匀，常温静置10min，于550nm处测定各管吸光值，分别记为A测定、A标准和A空白，计算ΔA测定=A测定-A空白，ΔA标准=A标准-A空白。空白管和标准管只需测1-2次。			

三、NO 含量计算

1. 按样本蛋白浓度计算

   NO含量（μmol/mg prot）= ΔA测定×（C标÷ΔA标准）×V样÷（V样×Cpr） = 0.025×ΔA测定÷ΔA标准÷Cpr

2. 按样本质量计算

   NO含量（μmol/g 质量）= ΔA测定×（C标÷ΔA标准）×V样÷（W×V样÷V样总） = 0.025×ΔA测定÷ΔA标准÷W

3. 按细菌/细胞数量计算

   NO含量（μmol/10^4 cell）=ΔA测定×（C标÷ΔA标准）×V样÷（V样×N÷V样总） = 0.025×ΔA测定÷ΔA标准÷N

4. 按液体体积计算

   NO含量（μmol/mL）= ΔA测定×（C标÷ΔA标准）×V样÷V样= 0.025×ΔA测定÷ΔA标准

C标：标准管浓度，0.025μmol/mL；V样：加入样本体积，0.24mL；V样总：加入提取液体积，1mL；Cpr：样本蛋白质浓度，mg/mL；W：样本质量，g ；N：细菌/细胞总数，以10^4计。

注意事项：

1、如果**样本匀浆液离心后上清仍旧浑浊**，可直接进行反应，反应后在 1mL 反应液中加入 250μL 澄清剂，混匀后静置 5min，离心后取 1mL 上清测定，**这种情况下需将空白管和标准管进行相同处理**。

2、如果ΔA 测定小于 0.01，可以增加样本量后再进行测定；如果ΔA 测定大于 0.8，建议将样本上清用提取液适当稀释后再进行测定。注意同步修改计算公式。

3、如果样本上清有颜色（在 550nm 下有吸收峰），则需要补测样本的对照管，即将显色液用相同体积的蒸馏水代替。在 550 nm 下测定吸光值 A，分别记为 A 标准、A 测定、A 空白、A 对照，计算 ΔA 标准=A 标准-A 空白，ΔA 测定=A 测定-A 对照。此时试剂盒规格为 50T/24S。

实验实例：

1. 取0.107g玉兰叶片样本，加入1mL提取液进行冰浴匀浆，离心后取上清，按照测定步骤操作，用1mL玻璃比色皿测得计算：ΔA测定=A测定-A空白=0.205-0=0.205，ΔA标准=A标准-A空白=0.364-0=0.364，按样本质量计算得：

   NO含量（μmol/g 质量）=0.025×ΔA测定÷ΔA标准÷W = 0.025×0.205÷0.364÷0.107=0.132 μmol/g 质量。

2. 取0.0868g小鼠心脏样本，加入1mL提取液进行冰浴匀浆，离心后取上清，按照测定步骤操作，用1mL玻璃比色皿测得计算：ΔA测定=A测定-A空白=0.212-0=0.212，ΔA标准=A标准-A空白=0.364-0=0.364，按样本质量计算得：

   NO含量（μmol/g 质量）=0.025×ΔA测定÷ΔA标准÷W = 0.025×0.212÷0.364÷0.0868=0.168 μmol/g 质量。

3. 取240μL牛血清样本，按照测定步骤操作，用1mL玻璃比色皿测得计算：ΔA测定=A测定-A空白=0.369-0=0.369，ΔA标准=A标准-A空白=0.364-0=0.364，按液体体积计算得：

   NO含量（μmol/mL）=0.025×ΔA测定÷ΔA标准 = 0.025×0.369÷0.364=0.025 μmol/mL。

相关发表文献：

[1] Peng X, Zhu L, Guo J, et al. Enhancing biocompatibility and neuronal anti-inflammatory activity of polymyxin B through conjugation with gellan gum[J]. International journal of biological macromolecules, 2020, 147: 734-740.

相关系列产品：

AC10081/AC10082 硝酸还原酶（NR）活性检测试剂盒

AC10319/AC10320	水土中亚硝酸盐含量检测试剂盒
AC10321/AC10322	食品中亚硝酸盐含量检测试剂盒
