# Supplementary material for: Screening potential anti-osteoarthritis compounds using molecular docking based on MAPK and NFκB pathways and validating their anti-osteoarthritis effect
Source: PLoS One. 2025 Mar 25;20(3):e0319686. doi: 10.1371/journal.pone.0319686 (PMC11936181; doi:10.1371/journal.pone.0319686)
Supplement: Attachment — Submitted filename: Response to Reviewers.docx [file pone.0319686.s010.docx]

We sincerely appreciate the comments of the four reviewers. They are very helpful to us.

**Reviewer #1**

**Comment:**

a. Protopine, 5-methoxyflavone, and 7,3',4'-trihydroxyisoflavone, which you mentioned, should also be subjected to 500-1000 nanosecond molecular dynamics simulation to investigate the stability of the interaction of the drug with the target protein using rmsd and hydrogen bond formation studies.

**Response:**

We are willing to use molecular dynamics simulation in future studies to explore the dynamic behaviors of molecules and evaluate the binding stability. However, the aim of this study is to screen potential anti-osteoarthritis compounds and verify their effects. We believe that, although not perfect, the computational and biological experiments in this study have achieved the aim.

**Comment:**

b. A list of catalytic site residues interacting with drugs and binding site pocket residues must be highlighted for each docking study.

**Response:**

The residues interacting with the drugs have been listed in Figure 10.

**Comment:**

c. A Github should be maintained to put all codes and input output files related to docking and molecular dynamics simulations.

**Response:**

Considering that access to Github is restricted in some countries, the related files have been put in the Supporting Information section.

**Comment:**

d. Figure numbers 4-8 are not clear, especially the diagrams at the bottom area; please replace these diagrams with high-resolution images.

**Response:**

These diagrams have been replaced with high-resolution images.

**Comment:**

e. Ligplot+ based ligand protein interacrion could also be used for better clarity.

**Response:**

Thank you for sharing your experience.

**Reviewer #2**

**Comment:**

1-The abstract should provide a concise summary of including key findings of the study.

**Response:**

A concise summary including key findings of the study has been provided.

**Comment:**

2-Authors suggest to add more recent citations with relevant studies (2023-2024).

**Response:**

More recent citations with relevant studies (2023-2024) have been added.

**Comment:**

3-The authors have to compare their results with literature data and improve this section completely.

**Response:**

The results have been compared with the literature data.

**Comment:**

4- Material and methods should include more details

**Response:**

More details have been included in the Materials and methods section.

**Comment:**

5- Why did the author choose the different proteins for docking studies? Give your appropriate reasons properly.

**Response:**

The reasons for choosing the different proteins for docking studies were given in the Introduction section and have also been given in the Selection of proteins and compounds for molecular docking section.

**Comment:**

6- How do authors validate the protein? Add Ramachandran plot.

**Response:**

The Ramachandran plots have been added.

**Comment:**

7- Enrich the discussion section by referencing other relevant studies that would be nice.

**Response:**

The discussion section has been enriched by referencing other relevant studies.

**Comment:**

8- I recommend adding the following five articles to the manuscript as citations.

https://doi.org/10.1016/j.chphi.2024.100753

https://doi.org/10.3390/molecules29143366

10.2174/0115734064304100240511112619

https://doi.org/10.1080/10286020.2024.2343821

https://doi.org/10.1016/j.jsps.2024.102062

**Response:**

These articles have been added as citations.

**Comment:**

9- References are not properly cited and should follow the journal style. The References section has to be revised after all corrections.

**Response:**

The References section has been revised.

**Comment:**

10- I suggest improving the English language.

**Response:**

The English language has been improved.

**Reviewer #3**

**Comment:**

Expand on the limitations of using human proteins for molecular docking while verifying effects on mouse chondrocytes. Although the authors acknowledge the species difference, a more detailed discussion of potential implications for translational relevance would be beneficial.

**Response:**

The expand on the limitations have been added in the Discussion section.

**Comment:**

Address the potential for off-target effects. The compounds selected may interact with other proteins beyond those targeted in this study. Discussing the possibility of off-target effects and strategies for mitigating them would enhance the study's rigor.

**Response:**

The possibility of off-target effects and strategies for mitigating them have been discussed in the Discussion section.

**Comment:**

While the chosen formula is reasonable, investigating other weighting approaches could provide additional insights and potentially identify different lead compounds.

**Response:**

Another weighting approach has been added in the Selection of compounds for drug-likeness, pharmacokinetics, bioactivity, and toxicity analysis, and cytotoxicity and anti-osteoarthritis effects testing section.

**Comment:**

Building upon the study's findings, outlining specific next steps for further research would enhance the paper's impact. This could involve testing the selected compounds in animal models of OA or exploring the development of novel compounds based on the identified binding identification.

**Response:**

Specific next steps for future research have been outlined.

**Comment:**

English proofreading and editing need to be further improved.

**Response:**

English proofreading and editing have been improved.

**Reviewer #4**

**Comment:**

The manuscript titled "Screening potential anti-osteoarthritis compounds using molecular docking based on MAPK and NFκB pathways and effect verification" presents data with significant potential. However, the order of presentation is chaotic, making it difficult to follow. The English is good, but the disorderly arrangement of the manuscript hinders comprehension. If I were a reader unfamiliar with this research, I would find it even more challenging to grasp the content.

**Response:**

The presentation has been modified. Many details have been added in the Materials and methods section to explain the order and content of the study design.

**Comment:**

The authors have omitted crucial details about the preparation of the computational section, making it difficult for readers to replicate the research. A more comprehensive description of this section is necessary for the manuscript to be fully understood and for the research to be reproducible.

**Response:**

The omitted crucial details about the preparation of the computational section have been added.

**Comment:**

The conclusion is weak and needs more improvement.

**Response:**

The conclusion has been improved.

**Comment:**

It is clear that this manuscript requires a comprehensive rewrite before it can be considered for another submission. The current structure and presentation of the data make it difficult for readers to understand the research.

**Response:**

This manuscript has been comprehensively rewritten.
